# Time-Dependent Toxicity and Health Effects Mechanism of Cadmium to Three Green Algae

**DOI:** 10.3390/ijerph191710974

**Published:** 2022-09-02

**Authors:** Lingyun Mo, Yilin Yang, Danna Zhao, Litang Qin, Baikang Yuan, Nan Liang

**Affiliations:** 1 College of Environmental Science and Engineering, Guilin University of Technology, Yanshan, Guilin 541006, China; 2 Guangxi Key Laboratory of Environmental Pollution Control Theory and Technology, Yanshan, Guilin 541006, China; 3 Technology Innovation Center for Mine Geological Environment Restoration Engineering in Southern Shishan Region, Ministry of Natural Resources, Xixiangtang, Nanning 530028, China; 4 Geological Environment Monitoring Station of the Guangxi Zhuang Autonomous Region, Xixiangtang, Nanning 530029, China

**Keywords:** cadmium, green alga, toxicity, antioxidant activity

## Abstract

As algae are extremely sensitive to heavy-metal ions and can be critical biological indicators in the heavy-metal toxicity analyses conducted by environmental health researchers, this paper explores the sensitivity to temporal toxicity of three species of green algae: *Scenedesmus obliquus*, *Chlorella pyrenoidosa*, and *Selenastrum capricornutum*. The method of time-dependent microplate toxicity analysis was used to systematically investigate the changes in the toxicities of the three green-algae species induced by different concentrations of cadmium (Cd). The chlorophyll *a* content, antioxidant enzyme activity, and malondialdehyde (MDA) content in the algae were analyzed to explore the mechanism of Cd toxicity after 96 h of exposure. The results showed that the toxic effects of Cd on the three algae species were time-dependent. By comparing the toxic effect of Cd, indicated by pEC_50_ (the negative logarithm of EC_50_), on the algae species at four durations of exposure (24, 48, 72, and 96 h), this study found that the indicator organisms had different sensitivities to Cd. The order of sensitivity was *C. pyrenoidosa* > *S. obliquus* > *S. capricornutum*. Cd exposure had significant effects on the chlorophyll *a* and MDA content and on the enzyme activity of superoxide dismutase (SOD) and catalase (CAT) in the algae species. The chlorophyll *a* content in the cells of the algae decreased with increasing Cd concentration. The enzyme activity of CAT and content of MDA increased with increasing Cd concentration, which indicated that Cd had an oxidative stress effect on the three algae species.

## 1. Introduction

Heavy metals are typical pollutants in the environment, and they have received global attention because they are hazardous to human health [1]. Heavy-metal pollutants are introduced into the water environment through various natural and artificial sources, including household emissions and industrial emissions such as mining and smelting [2]. Heavy metals are persistent, nonbiodegradable pollutants that easily accumulate in organisms, posing a threat to ecosystems and human health. For example, in Islam et al. [3], levels of six metals in three fish species from three urban rivers in Bangladesh were measured; the results showed that As and Pb in muscle were particularly hazardous and potentially risky for the fish consumer in Bangladesh. The heavy metal cadmium (Cd) has entered the water environment due to its extensive application in production and daily life and has become an environmental pollutant. Aquatic organisms have a strong absorbability for Cd, which accumulates easily in organisms. High concentrations of pollutants can impair human health by causing oxidative damage to biological tissues [4,5,6].

Prolonged exposure to Cd poses serious threats to environmental health. Moreover, the exposure time and exposure concentration in the natural environment affects the toxicities of pollutants [7]. Xiong et al. [8] demonstrated that the toxic effect of sulfa antibiotics on *Scenedesmus obliquus* increased with the prolongation of exposure time. Zhang et al. [9] found that the toxicities of the heavy metals Cd, Pb, and Mn increased with the prolongation of exposure time. Therefore, in addition to investigating concentration levels, studies of pollutant toxicity examine exposure time.

Algae are extremely sensitive to heavy-metal ions and can serve as critical biological indicators for heavy-metal-toxicity analysis in environment health research [10]. However, the same heavy metal will affect the growth and metabolism of algal cells to different degrees for different biological indicators. In the *Nostoc entophytum*, cadmium exposure to cells causes destruction of some pigment–protein complexes, and changes in catalase activity, lipid peroxidation, membrane damage, and protective mechanisms could be found [11]. Cd could induce an extreme shrinkage and faint appearance change of the thylakoid membrane system in *Chlorella variabilis* [12]. *Scenedesmus obliquus*, *Chlorella pyrenoidosa*, and *Selenastrum capricornutum*, which belong to the *Chlorophyta phylum*, are free unicellular algae that reproduce rapidly and hard to settle and exhibit a uniform distribution. These Chlorophyta species can be fully contacted with pollutants and are easy to be cultivated, making them ideal biological indicators in toxicity analysis for health effects.

In order to study the time-dependent Cd toxicities and Cd sensitivities of different species of green algae, the species *S. obliquus*, *C. pyrenoidosa*, and *S. capricornutum* were used as biological indicators. Time-dependent microplate toxicity analysis (T-MTA) was used [13,14] to determine the growth inhibition, chlorophyll *a* content, catalase (CAT) activity, superoxide dismutase (SOD) activity, and malonaldehyde (MDA) content in these algae species at different exposure times. The results of this analysis may provide a data reference for co-environmental protection and ecological health assessments that focus on Cd contaminations.

## 2. Materials and Methods

The Cd (CdCl_2_•2.5H_2_O, CAS 7790-78-5, molecular weight = 228.36, purity > 99.0%) was purchased from Damas-Beta Co., Ltd. (Shanghai, China). The stock solution of Cd was prepared with Milli-Q water and stored at 4 °C. The concentration of the CdCl_2_•2.5H_2_O stock solution was 4.46 mM.

*S. obliquus* (FACHB-12), *C. pyrenoidosa* (FACHB-5), and *S. capricornutum* (FACHB-271) were purchased from Institute of Hydrobiology, Chinese Academy of Sciences, and cultivated with medium formulation and culture procedure as described in the literature [15].

The data for Cd’s toxicity to *S. obliquus*, *C. pyrenoidosa*, and *S. capricornutum* were determined by T-MTA [11,12]. Seven concentration series in 3 parallels and 14 controls were arranged in a transparent 96-well microplate (Turner BioSystems Inc., N Mary Ave, Sunnyvale, CA, USA) and then cultured at 25 ± 1 °C, 3000 lux, 12 h of light, and 12 h of darkness. The final Cd concentrations in the serial dilutions were in the order *S. obliquus*: 9.10 × 10^−4^, 1.70 × 10^−3^, 3.00 × 10^−3^, 4.50 × 10^−3^, 7.60 × 10^−3^, 1.40 × 10^−2^, and 2.30 × 10^−2^ mM; *C. pyrenoidosa* and *S. capricornutum*: 1.30 × 10^−^^3^, 1.80 × 10^−^^3^, 2.50 × 10^−^^3^, 3.60 × 10^−^^3^, 5.40 × 10^−^^3^, 7.20 × 10^−^^3^, and 1.10 × 10^−^^2^ mM. The optical density (OD) at 690 nm of *S. obliquus*, *C. pyrenoidosa*, and *S. capricornutum* in the microplate were determined with the Power Wave microplate spectrophotometer (American BIO-TEK Company, Inooski, VT, USA) at 24, 48, 72, and 96 h of exposure. The procedures described above were repeated three times. The toxicity of Cd is expressed in terms of inhibition rate (*E*), and the formula is given in Equation (1).

(1) $$E=\left(1-\frac{\mathrm{OD}}{{\mathrm{OD}}_{0}}\right)\times 100\%,$$

where OD denotes the average optical density of the experimental group, OD_0_ denotes the average optical density of the control group.

To quantitatively describe the toxicity effects, the concentration–effect curves (CRCs) were fitted using the Weibull function (Equation (2)) [16].

(2) $$E=1-exp\;(exp\;(\alpha +\beta \times \log_{10}\left(c\right))),$$

where *α* and *β* refer to the location and slope parameters, respectively, *E* (0 ≤ *E* ≤ 1) refers to a chemical’s effect and *c* refers to a chemical’s concentration.

The chlorophyll *a* content, CAT activity, SOD activity, and MDA content of *S. obliquus*, *C. pyrenoidosa*, and *S. capricornutum* were determined at exposure times for 96 h. Chlorophyll *a* content was determined using chlorophyll *a* fluorescence, and CAT activity, SOD activity, and MDA content were determined using test kits (Nanjing Jiancheng Institute of Biological Engineering., Nanjing, China) [17,18].

15 mL of algal was taken into a 50 mL round-bottom centrifuge tube and centrifuged for 10 min at 10,000 r/min. With supernatants deserted, we added 15 mL of 95% ethanol solution, shook well and then sonicated for 20 min in an ice bath. Extracted at 4 °C for 24 h in the dark, after extraction, the tubes were centrifuged at 10,000 r/min for 10 min. The absorbance of the supernatant was measured at 665 nm and 649 nm in a quartz cuvette of 1 cm optical diameter, using 95% ethanol as a reference, and the formula is given in Equation (3).

(3) $$C_{a}=13.95A_{665}-6.88A_{649},$$

The enzyme activity was determined by referring to the method of Yu et al. [17] and Bian et al. [18]. However, in the experiment, we found that the enzyme activity obtained in the original method when 2 mL of algae was low, and the error was significant. Therefore, we improved the experimental method by taking 5 mL of green algae collected from each conical flask to a centrifuge tube at 96 h, and centrifuged at 10,000 r/min for 10 min (20 °C). With supernatants deserted, green algae in the sediment were re-suspended in ice-cold phosphate-buffered saline (PBS, 0.1 M, pH 7), transferred into 1.5 mL centrifuge tubes, sonicated for 15 min in a water bath at 4 °C and 10,000 r/min centrifugation for 10 min (4 °C), and the supernatant was taken to measure CAT activity, SOD activity, and MDA content.

Each experiment was conducted at least triplicate and the results were expressed as mean ± SEM. Significant differences (*p* < 0.05) were statistically estimated using one-way ANOVA of Duncan multiple comparisons and are indicated using different letters (a, b, c, d, e, and f).

## 3. Results and Discussion

### 3.1. Trial on the Time-Dependent Toxicity of Cd to the Three Green-Algae Species

Table 1 presents a comparison of the concentration–effect parameters at different exposure times based on results calculated by the nonlinear least-squares fitting method and the regression coefficients (position parameter *α* and slope *β*). As the table shows, the toxicity of Cd gradually increased with the extension of exposure time, clearly demonstrating the time-dependent nature of the toxicity effects. These results indicated that the indicator organisms had different sensitivities to Cd. According to the *pEC*_50_ values for the three algae species at four exposure times, the indicator organisms had different sensitivities to Cd, and the sensitivities were ranked as follows: *C. pyrenoidosa* > *S. obliquus* > *S. capricornutum*.

**Figure 1:**
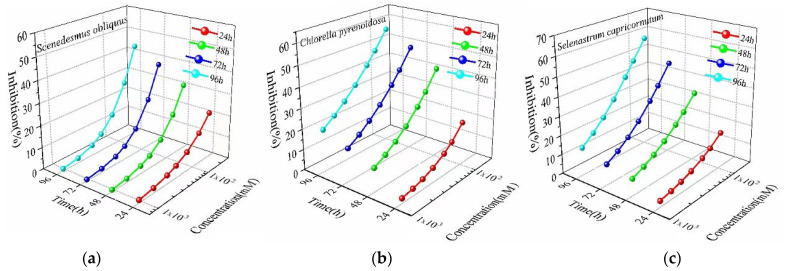
Concentration–effect curves of *S. obliquus* (**a**), *C. pyrenoidosa* (**b**) and *S. capricornutum* (**c**) at different durations of Cd exposure.

In line with the nonlinear least-squares method, CRC values were obtained through the fitting. As Figure 1 shows, the growth inhibition rates of Cd in the three algae species increased with increasing exposure time. Cd from 9.06 × 10^−^^4^ to 2.26 × 10^−^^2^ mM, toxicity to *S. obliquus* rose from 20.84% to 42.97% when Cd extended exposure time from 24 to 96 h. Likewise, for *C. pyrenoidosa* and *S. capricornutum*, the toxicity values increased with the extension of exposure time, but the toxicity effects were significantly different. Table 1 and Figure 1 show that Cd had noticeable time-dependent toxicity effects on different indicator organisms.

### 3.2. Mechanism of Cd Toxicity to the Three Green-Algae Species

**Figure 2:**
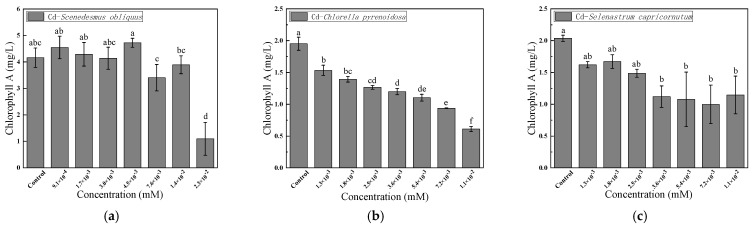
Effects of 96-h Cd exposure on the content of chlorophyll *a* in *S. obliquus* (**a**), *C. pyrenoidosa* (**b**), and *S. capricornutum* (**c**). The different letters: a, b, c, d, e, and f in the figure indicated that significant differences (*p* < 0.05).

Cd toxicity was most significant at 96 h. Therefore, algae with an exposure time of 96 h were selected for mechanistic analysis to better reflect the mechanism of Cd toxicity. Figure 2 shows the changes in chlorophyll *a* content in the three algae species after 96 h of exposure to different Cd concentrations. For *S. obliquus*, the chlorophyll *a* content was 9.35% and 13.54% higher than that of the control when the Cd concentration was 9.1 × 10^−^^4^ and 4.5 × 10^−^^3^ mM, respectively. At Cd concentrations of 2.3 × 10^−^^2^ mM, the chlorophyll *a* content was significantly lower than that of the control (*p* < 0.05), indicating that chlorophyll *a* synthesis in *S. obliquus* was inhibited and manifested as a decrease in chlorophyll *a* content. Different Cd concentrations could inhibit the synthesis of chlorophyll *a* in *C. pyrenoidosa* and *S. capricornutum*, and there was a significant difference between the control group and the high concentration experimental group (*p* < 0.05). When the concentration of Cd was 1.3 × 10^−^^3^ mM, the chlorophyll *a* content of these two algae species was 20.00% lower than that of the corresponding control groups. When the concentration of Cd was 1.1 × 10^−^^2^ mM, the chlorophyll *a* content of these two algae species was 68.66% and 43.75% lower than that of the corresponding controls, respectively. It is noteworthy that there are also significant differences between the experimental groups (*p* < 0.05), as can be clearly seen in Figure 2b. These results showed that the growth inhibition rate was increased in correlation to decreasing chlorophyll content. In other words, the changes in the content of chlorophyll *a* were consistent with the changes in the ecotoxicity effects.

Nevertheless, chlorophyll is a ubiquitous natural pigment in phytoplankton. It is an essential index of plants’ light-utilization ability in assessments of the photosynthetic physiological capacity of plants and the degree of environmental stress [19]. Therefore, in this study, the decreased content of chlorophyll *a* reflected Cd’s inhibition of the growth and metabolism of the three algae species. Kovacik et al. [20] reported that the content of chlorophyll *a* in *Selenastrum quadricauda* decreased under Cu stress. In addition, Ismaiel et al. [21]. and Shivaji et al. [22]. found that the addition of Cd impeded the growth of *Pseudochlorella pringsheimii* MIYA 102 and *S. obliquus* and decreased their chlorophyll content. The main reason is that chlorophyll content is often used as an indicator of algal toxicity [23]. The process by which algae adsorb heavy metals comprises two stages: extracellular adsorption and intracellular enrichment [24]. Therefore, it is generally believed that heavy metals inhibit chlorophyll synthesis by inhibiting the activities of enzymes related to chlorophyll synthesis [25].

**Figure 3:**
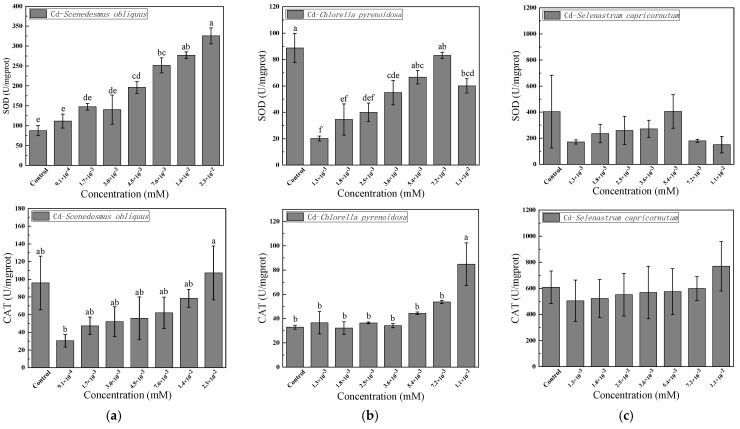
Effects of 96-h Cd exposure on the activity of superoxide dismutase (SOD) and catalase (CAT) in *S. obliquus* (**a**), *C. pyrenoidosa* (**b**), and *S. capricornutum* (**c**). The different letters: a, b, c, d, e, and f in the figure indicated that significant differences (*p* < 0.05).

The effects of 96-h Cd exposure on SOD and CAT enzyme activity in the cells of *S. obliquus*, *C. pyrenoidosa* and *S. capricornutum* under different Cd concentrations are shown in Figure 3. As the figure shows, in *S. obliquus*, SOD enzyme activity increased with increasing Cd concentration, up to 3.66 times that of the control group. Moreover, the experimental and control groups were significantly different in terms of SOD enzyme activity (*p* < 0.05). In *C. pyrenoidosa*, SOD enzyme activity initially increased and then declined. For this species, SOD enzyme activity reached its minimum value when the concentration of Cd reached its minimum. However, at the highest level of SOD activity, the differences between the experimental and control groups were not significant, whereas the differences at other levels of SOD activity were significant (*p* < 0.05). Unlike other species, there was no statistically significant change in SOD enzyme activity with concentration of Cd in *S. capricornutum*.

As shown in Figure 3, when the Cd concentration was in the range of 9.1 × 10^−^^4^ to 2.3 × 10^−^^2^ mM, the CAT enzyme activity of *S. obliquus* gradually grew. The difference in CAT enzyme activity between the experimental and control groups of *S. obliquus* was significant (*p* < 0.05) when the Cd concentration was at its minimum. With increasing Cd concentration, the CAT enzyme activity of *C. pyrenoidosa* increased. When the Cd concentration reaches a maximum value (1.1 × 10^−^^2^ mM), the CAT enzyme activity of *C. pyrenoidosa* reached its highest level and was 3.71 times that of the control group, and the differences between both groups were significant (*p* < 0.05). There were no statistically significant changes in CAT enzyme activity of *S. capricornutum* that were produced with changes in Cd concentration.

The SOD–CAT system is a cell’s first line of defense against oxidative poisoning [26,27], and SOD is an essential peroxidase that uses free radicals as a substrate. The primary function is to catalyze the production of high-toxicity $O_{2}^{-}$ and low-toxicity H_2_O_2_. CAT can catalyze the decomposition of H_2_O_2_ into O_2_ and H_2_O, mitigating the oxidation hazards caused by H_2_O_2_. SOD and CAT represent the oxidation and antioxidation system, which is an indispensable process by which organisms resist the damage caused by oxygen free radicals [28]. In this study, when Cd was exposed to the fluid of three algae species, the changes in SOD and CAT enzyme activities were different, which may have been due to Cd’s other oxidative stress effects on the cells of different algae species [29]. Kurama et al. [30] demonstrated that the production of a large amount of SOD is a mechanism by which plants protect chloroplasts from damage by organic pollutants.

An increase in SOD enzyme activity causes more superoxide to be converted into H_2_O_2_, which in turn activates the removal of excess H_2_O_2_ in the cell by CAT enzyme activity [31]. Therefore, an increase in CAT enzyme activity can be regarded as an adaptive strategy that protects the cell against the oxidant effect. Under the influence of Cd, the enzyme-activity change sequence corresponding to the concentration of SOD and CAT indicated by EC_50_ was *C. pyrenoidosa* < *S. obliquus* < *S. capricornutum*.

**Figure 4:**
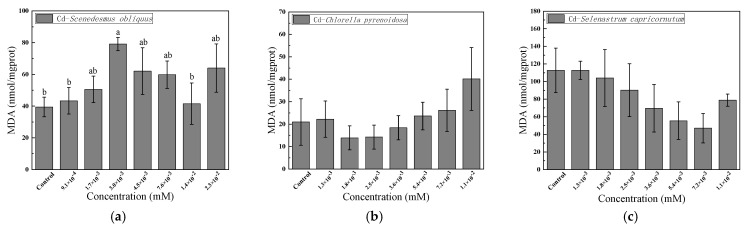
Effects of 96-h Cd exposure on the content of malondialdehyde (MDA) in *S. obliquus* (**a**), *C. pyrenoidosa* (**b**) and *S. capricornutum* (**c**). The different letters: a, and b in the figure indicated that significant differences (*p* < 0.05).

Figure 4 shows the effects on the MDA content of *S.* *obliquus, C. pyrenoidosa*, and *S. capricornutum* under exposure to different concentrations of Cd for 96 h. As the figure shows, the MDA content of *S.* *obliquus* initially increased, then decreased, and finally increased with increasing Cd concentration. When the Cd concentration was 3.0 × 10^−^^3^ mM, the MDA content was twice that of the blank control, and when the Cd concentration was 1.4 × 10^−^^2^ mM, the MDA content of *S.* *obliquus* was 1.1 times that of the blank control. The MDA content of *C. pyrenoidosa* increased, reaching more than twice that of the blank control. With increasing Cd concentration, the MDA content of *S. capricornutum* initially decreased and then increased.

MDA is a peroxidation product of fatty acids in membrane lipids, and it is an important index of lipid peroxidation. It is commonly used to reflect the peroxidation damage caused by environmental-pollution stress to algal cells [32,33]. Khazri et al. [34] showed that when a filamentous blue-green alga was exposed to Cd, the content of MDA in the plant’s cells increased significantly. This finding was consistent with the present study’s results for *S.* *obliquus* and *C. pyrenoidosa*. This indicates that the increase in SOD and CAT activities induced by Cd was insufficient to scavenge oxidative free radicals in cells, and Cd produces oxidative stress on algal cells, leading to the accumulation of MDA and thus affecting the integrity of the cell membrane. The growth and physiological and biochemical functions of the algal cells were affected. The changing trend of MDA content was consistent with the changing trend of SOD and CAT. The MDA content of algal cells exposed to Cd indicated by EC_50_ follows the order: *Chlorella pyrenoidosa* < *Scenedesmus obliquus* < *Selenastrum capricornutum*.

## 4. Conclusions

The results demonstrated that the toxicity effects of Cd increased with the extension of exposure time, and the time–concentration–effect relationships were apparent. In addition, the toxicities of three indicator organisms (*S. obliquus, C. pyrenoidosa*, and *S. capricornutum*) that were exposed to Cd at the same time were different. This confirmed that the sensitivities of various indicator organisms to the effects of Cd were distinct. The indicator organisms were found to have different sensitivities to the heavy metal Cd, which follow the order: *Chlorella pyrenoidosa* > *Scenedesmus obliquus* > *Selenastrum capricornutum*.

Under 96 h of exposure, the stress damage caused by different concentrations of Cd to the three species of green algae manifested in oxidative stress. The results showed that the content of chlorophyll *a* in algal cells decreased with increasing Cd concentration, mainly because the algal cells could not obtain enough energy to maintain their growth, which led to the inhibition of algal cell growth. In addition, the SOD, CAT, and MDA content of the algal cells exposed to Cd EC_50_ concentration was as follows: *C. pyrenoidosa* < *S. obliquus < S. capricornutum*. The order of sensitivity of the corresponding algal cells was opposite to that of the corresponding algal cells: *C. pyrenoidosa* > *S. obliquus* > *S. capricornutum*. It can be concluded that in the unfavorable growth environment, the active-oxygen-scavenging antioxidant defense systems of the algal cells were triggered to activate antioxidases (SOD and CAT) to maintain a relatively stable active oxygen metabolism.

## Figures and Tables

**Table 1:** *α* and *β* values of the Weibull functions, fitting results (*R*^2^ and *RMSE* values), *EC_50_* values, *pEC_50_* values, and other parameters for Cd at four time points.

Algae	Exposure Time /h	Model Parameters				*EC*_50_ (mM)	*pEC*_50_
		*α*	*β*	*R* ^2^	*RMSE*		
*Scenedesmus obliquus*	24	9.20	2.13	0.9893	0.0108	4.79 × 10^−2^	4.32
	48	13.02	2.80	0.9873	0.0185	2.24 × 10^−2^	4.65
	72	15.00	3.14	0.9946	0.0255	1.67 × 10^−2^	4.93
	96	15.00	3.09	0.9937	0.0228	7.83 × 10^−3^	5.11
*Chlorella pyrenoidosa*	24	10.31	2.37	0.9718	0.0143	3.13 × 10^−2^	4.50
	48	7.58	1.64	0.9862	0.0176	1.43 × 10^−2^	4.85
	72	7.05	1.49	0.9744	0.0264	1.05 × 10^−2^	4.98
	96	6.68	1.38	0.9787	0.0255	7.66 × 10^−3^	5.12
*Selenastrum capricornutum*	24	8.71	2.05	0.9756	0.0122	5.64 × 10^−2^	4.25
	48	8.65	1.86	0.9886	0.0141	2.63 × 10^−2^	4.63
	72	10.70	2.17	0.9875	0.0206	1.67 × 10^−2^	4.78
	96	11.51	2.25	0.9874	0.0237	1.40 × 10^−2^	4.85

## Data Availability

Not applicable.
